# JANE: efficient mapping of prokaryotic ESTs and variable length sequence reads on related template genomes

**DOI:** 10.1186/1471-2105-10-391

**Published:** 2009-11-29

**Authors:** Chunguang Liang, Alexander Schmid, María José López-Sánchez, Andres Moya, Roy Gross, Jörg Bernhardt, Thomas Dandekar

**Affiliations:** 1 Department of Bioinformatics, Biocenter, University of Würzburg, Am Hubland, D-97074 Würzburg, Germany; 2 Department of Evolutionary Genetics, Institut Cavanilles de Biodiversitat i Biologia Evolutiva, University of Valencia, Spain; 3 Department of Microbiology, Biocenter, University of Würzburg, Am Hubland, D-97074 Würzburg, Germany; 4 Institute for Microbiology, Ernst-Moritz-Arndt-University Greifswald, Jahnstrasse 15, 17487 Greifswald, Germany; 5 EMBL, Postbox 102209, D-69012 Heidelberg, Germany

## Abstract

**Background:**

ESTs or variable sequence reads can be available in prokaryotic studies well before a complete genome is known. Use cases include (i) transcriptome studies or (ii) single cell sequencing of bacteria. Without suitable software their further analysis and mapping would have to await finalization of the corresponding genome.

**Results:**

The tool JANE rapidly maps ESTs or variable sequence reads in prokaryotic sequencing and transcriptome efforts to related template genomes. It provides an easy-to-use graphics interface for information retrieval and a toolkit for EST or nucleotide sequence function prediction. Furthermore, we developed for rapid mapping an enhanced sequence alignment algorithm which reassembles and evaluates high scoring pairs provided from the BLAST algorithm. Rapid assembly on and replacement of the template genome by sequence reads or mapped ESTs is achieved. This is illustrated (i) by data from Staphylococci as well as from a *Blattabacteria *sequencing effort, (ii) mapping single cell sequencing reads is shown for poribacteria to sister phylum representative *Rhodopirellula Baltica* SH1. The algorithm has been implemented in a web-server accessible at http://jane.bioapps.biozentrum.uni-wuerzburg.de.

**Conclusion:**

Rapid prokaryotic EST mapping or mapping of sequence reads is achieved applying JANE even without knowing the cognate genome sequence.

## Background

### Problem

In eukaryotes, mapping of eukaryotic ESTs (expressed sequence tags) to DNA has to deal with splicing, widely distributed parts of genome sequence have to be aligned and the genome sequence is generally known. In contrast, JANE deals with the opposite problem: Prokaryotic ESTs or variable sequence reads are mapped, assigned and analyzed in a sequencing project well before the prokaryotic genome sequence is completely known. In particular rapid EST sequencing (e.g. this study and [1]), ecological community sequencing [2,3] and single cell sequencing [4,5] provide large data sets in prokaryotes though the genome sequence is not or only very partially known. For these use cases JANE (Just Analyze Nucleotides and ESTs) allows (i) to rapidly identify the function of ESTs as well as short sequence reads, (ii) to map ESTs and variable reads (multiple fasta-format files) to an already known related prokaryotic genome and (iii) to reconstruct a "virtual genome" of the unknown or incomplete prokaryotic genome already before assembly of a new prokaryotic genome including prediction of badly sampled regions. (iv) As prokaryotic cDNAs reflect multigene transcription units, JANE's rapid EST mapping can be used for operon mapping. (v) ESTs from clinical isolates (e.g. different *S. aureus *strains) can be rapidly mapped to related known genomes. (vi) Mapped reads are statistically analyzed, e.g. to show highly transcribed regions in the genome or undersampling as well as repeat regions. (vii) Any other type of short sequences can be mapped to the chosen template genome. In particular, this speeds up genome predictions in single cell sequencing efforts and from ultrafast transcriptome sequencing efforts, e.g. pyrosequencing reads from sequencing of cDNA libraries.

Data sets and use cases for JANE are: Use-case (i) transcriptome data (ESTs, mRNA, cDNA) to map to a genome template not identical to the transcriptome that is investigated as the genome template is not known. Use-case (ii) single cell sequencing data and the use case is here to predict or establish a more complete genome sequence. In contrast, for ultrafast sequencing recent developments include ultrafast DNA sequencing assembly programs such as Maq [6], SOAP [7], SeqMap [8] and Bowtie [9] and RMAP [10] which are optimal to map short and very short reads to their cognate genome. This is the ultrafast sequencing use-case (iii) with read lengths from 36-400 bp which are then assembled or mapped to their cognate DNA template. JANE is compared also to this software.

### Applications

We show JANE's good performance in JANE's standard use cases (i, ii), that is in particular for assembling variable sequence reads (from few basepairs to kilobases) in mapping to a related, non-identical template genome in the tasks mentioned above as described in detail in [1-5]. Here mapping should be efficiently done without knowing the exact DNA sequence. However, then it is difficult to accurately map the variable (short, long) sequence reads as there are no perfect matches and if standard sequence comparison algorithms are used, the search may not find any matches or mapping location and range of EST is frequently ambiguous. This problem is solved in JANE by a specific assembly algorithm for HSPs and start alignments. Moreover, the function of the EST or mapped region should be predicted. Furthermore, the template genome used for the mapping should be stepwise replaced by the contigs achieved after mapping a sufficient number of ESTs or short sequence reads and an overview on the not assigned sequences obtained. We developed for these problems JANE as a user-friendly application. It includes a new implemented harvesting program for extension and assembly of HSPs. HSPs are high scoring pairs of two sequence fragments of arbitrary but equal length whose alignment is locally maximal and for which the alignment score meets or exceeds a threshold or cutoff score. The HSPs were collected before by a parameter adapted BLAST. Our focus is in the following on application aspects of the JANE software in its standard use cases, we do not give an in depth treatment of sequence alignment methods, for this the reader is instead referred to recent reviews on the topic such as [11].

Besides mapping prokaryotic ESTs, JANE is useful for instance in the following scenarios: Mapping in an ongoing genome sequencing effort where no genome sequence is available yet a number of ESTs is already there (the *Blattabacteria *project shown in the following is such an example from own work), analysis and mapping of ESTs from clinical isolates where no genome sequence is available (e.g. clinical Staphylococci isolates) as well as rapid mapping in transcriptomics studies without a matching genome sequencing effort (for instance regarding different Blochmannia strains [12]). Furthermore, in single cell sequencing efforts (a new technology to look at bacteria non-cultivable in environmental samples) the situation occurs quite often that incomplete reads are only available, the genome sequence is not known and mapping to a template genome is useful or required [5].

## Implementation

The program has been written in Perl using bioperl and GD graphics libraries, the visualization interface was implemented with Javascripts. JANE is currently running on an Apache server, with a PostgreSQL http://www.postgresql.org/ database support.

### Algorithm

In JANE, a specific algorithm assembles HSPs (alignment and assembly of high scoring pairs of similarity regions between a sequence query and a similar database entry) such that EST mapping to a template genome is optimized. The JANE algorithm does this task differently and better than BLAST [13-15] (see results below). For the step before, the HSP generation, the BLAST program package was applied and the HSP generation parameters optimized. The combination allows mapping of ESTs and variable sequence reads even to distant template genomes.

#### Initial HSP extraction

**Figure 1:**
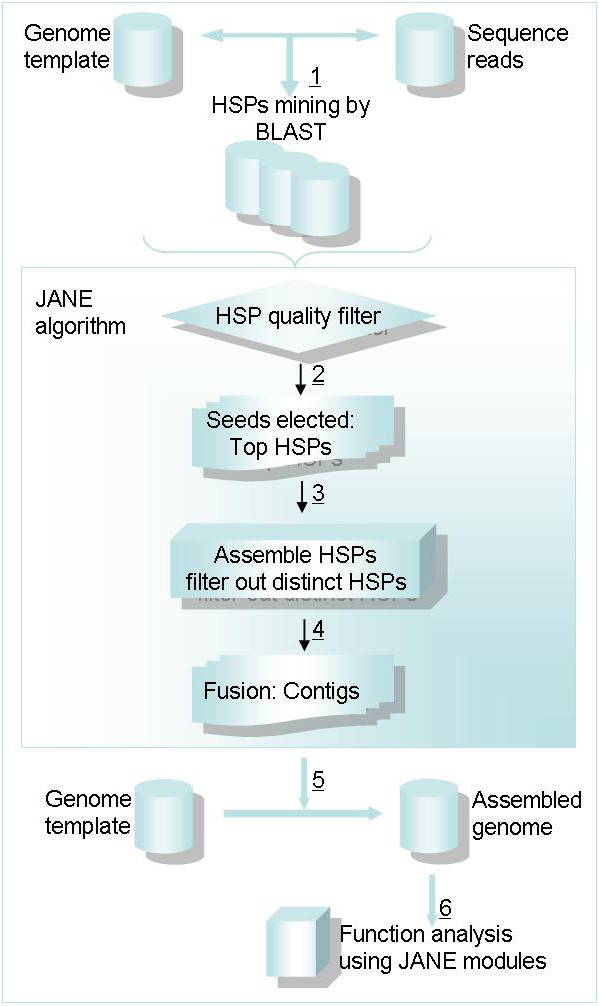
**Strategy of JANE**. 1) HSP regions (high scoring pairs) are collected applying the BLAST algorithm. Parameters were optimized to detect alignments with lower sequence similarity. 2) HSP fragments are processed using JANE's algorithm, a quality filter is applied to increase accuracy. 3) Top scoring HSPs for each reads are used as anchors. Next, further HSPs are selectively considered (only the closest first) if not too distant (criterion: remaining length of the EST/sequence read to be mapped). 4) The algorithm assembles and calculates potential coverage. Two reads sharing overlapping regions are consecutively merged, forming a contig. 5) A predicted genome is generated replacing more and more parts from the template genome by mapped and assembled sequences. 6) Modules in the JANE toolkit predict function information on individual ESTs or sequence reads.

Here the BLAST package (version 2.2.15) was applied and parameters for lower-restriction searches applied (Fig. 1). Parameters included a lower penalty (-1) for a nucleotide mismatch compared to the default value (-3), an E-value of 0.1 for harvested ESTs by BLAST and the low-complexity filter is disabled. This reduces the accuracy but significantly enhances the harvest sensitivity. Moreover, this allows longer extension both in 3' and 5' direction of the EST paired region. It is critical for aligning EST to a relatively distant genome template, e.g., *Blattabacteria *to *Candidatus Sulcia muelleri *GWSS [GenBank: CP000770]. In this example, a search applying BLAST and its standard parameters can only locate about a seventh of the hits, not sufficient for an efficient mapping. Furthermore, we found that for best results in EST mapping (maximum number of correct mapped ESTs) the low-complexity filter (DUST) should be disabled, since filtering for low-complexity regions strongly reduces the available HSPs that are subsequently evaluated. This disadvantage far outweighs the advantageous reduction in compositional biased sequences by the filter.

#### Pairwise alignment and assembly algorithm

Here BLAST was no longer used, instead a new program was written for a maximum use of revealed sequence co-linearity. The top HSPs of each read according to their scores are sorted and used as start alignments in the following step. Only the top scoring HSP in a database entry provides a start alignment for anchoring the alignment. Nearby each start alignment, the remaining unmatched regions are in general located either in the UTRs or in less well conserved regions. The start alignment provides a strong signal. Its position and in the considered read or EST suggests a potential coverage region in the corresponding genome. Based on this, further HSPs of lower score are considered if they are still in the remaining EST or sequence read region (distance criterion). These secondary HSPs still carry useful information for the direction of further alignments and improve the coverage in the final mapping. Moreover, the utilization of these secondary HSPs contributes to overcome the obstacles derived from length variation of non-translated regions and their less well conservation and it improves mapping of distantly related sequence reads to a template. For optimal results, the alignment penalty in these secondary alignment regions is set to zero both for gap creation and extension.

The assembly of anchoring and secondary HSPs results in a series of local co-linear islands. Next the program assembles the islands if any two in the template are overlapping and such steps are repeated in both directions, forming a contig region. If JANE is used in a large-scale sequencing project allowing high coverage of different genome regions, the regions of the template genome which are never hit by sequence reads are with high probability insertions in the template genome respective to the new sequenced genome (this can be directly checked by the user applying JANE). However, this shows also that the requirements for the template genome are easy to fulfill: A moderate overall sequence similarity (over 60% amino acid identity in household enzymes, see below) to the organism the ESTs are derived from and a triple coverage of the template genome with typical Sanger or 454 sequencing reads (400 bp reads) are sufficient.

#### Filter for higher alignment accuracy

JANE considers next many secondary HSPs after a major HSP for anchoring the alignment has been identified. For higher specificity, a filter in JANE's algorithm discards all HSPs of extremely-low identity (<20%). From the HSPs harvested, only the HSP with the top score for each read is considered and used as anchor for the first alignment iteration. Further HSPs are subsequently considered. Only the closest one is connected with the anchor region when the distance from the anchor is not too far (distance cutoff criterion: distance less or equal to the remaining length of the EST which is considered). The assembly is iterated and always carried out from the anchor region to the closest HSP candidate from the complete harvested HSPs. Not joined HSPs are filtered out during the procedure.

#### Change of parameters and program modules

JANE's web-interface allows users to specify the following parameters: E-Value, minimum HSP length (default setting: 70) for HSP harvests as well as a zoom factor for optimal visualization. Within the program, a standaloneBlast routine is used exclusively to generate HSPs. In this task it enables many parameters (more then 20 different parameters; set as default for later processing by JANE according to the default setting used in Blast) to be modified by an interested programmer. Next, JANE's HSP harvesting program is implemented. It is integrated with and assembles the bioperl StandaloneBlast generated HSPs. All specific parameters of JANE including top HSPs considered, filtering criteria, alignment parameters can be freely varied in its code. For instance, one can use different filter criteria or modify the distance cutoff for HSPs to be considered in an EST mapping task. This can be interesting for further applications or modified sequence similarity searches or comparisons. Note however, that all parameters have been optimized in their default setting by us for the intended prokaryotic sequence read mapping task to a not perfect template genome.

#### Strategy

JANE's strategy is an elective HSP assembly strategy (Fig. 1). As a comparison example, DIALIGN and its successor DIALGN-TX [16,17] enable improved multiple sequence alignments with a remarkable higher speed in comparison to the popular global alignment application CLUSTALX. DIALIGN uses a greedy strategy for multiple alignment, all possible pairs of input sequences are taken. DIALIGN-TX uses instead a guide tree based on pairwise similarity scores, and considers as DIALIGN-T not only the weight scores of individual fragments (overlap weights) but also the overall degree of similarity between the two sequences involved in the fragment. A fragment is here a pair of two equal lengths segments from two different input sequences, a local pairwise gap-free alignment of these two sequences. The strategy of JANE is different: Instead of multiple-alignments, JANE implements an algorithm to have multiple fragments assembled and mapped to one reference template. Furthermore, JANE considers for each sequence read to be mapped only the top HSP as anchor and then the closest neighbour HSP if it is still within EST/sequence read length. Iteratively further HSPs are anchored in this way. By this strategy the program can cope with high sequence diversity and diverse read lengths (from bp to kb) and can deal with not close related template genomes in mapping. In comparison to BLAST [14] the HSP assembly strategy is also improved regarding the specific task of mapping to a non-identical template. In particular, JANE starts only with the top scoring HSP in any alignment for anchoring and secondary HSPs are only assembled if they are the closest neighbour and within the length of the individual sequence read to be mapped. In summary, JANE accepts large sequence diversity and read length (from bp to kb) and thus can efficiently map variable reads from different sequencing approaches to similar and dissimilar genome templates. Its mapping strategy is particularly advantageous in transcriptome sequencing and single cell sequencing efforts when the complete genome sequence is not available.

#### Mapping visualization and virtual genome

A user-friendly interface facilitates to retrieve mapping information and alignment figures. Moreover, a tab-delimited alignment profile can be downloaded from the results page for further analysis using third-party software, e.g., GNU R. In growing contig-regions the template genome is step by step replaced by mapped ESTs or short sequence reads and the new genome thus takes over. Capital letters record assembled reads whereas lowercase letters denote the used template genome. The resulting file can be downloaded for further analysis.

#### Function analysis

JANE provides a toolkit for rapid function assignment [see additional file 1: Fig. S1]. Novel sequences can be searched against a database derived from the COG/KOG collection [18], the program "COGmaster" will generate a table listing the putative COG classification and description. In contrast to cognitor [18], our module enables batch-searches both for long nucleotide and protein sequences. This helps to rapidly obtain a raw annotation, e.g., for a genomic fragment.

### Further applications contained in the JANE package

A "Format converter" assists in fast sequence format conversion (12 formats) in particular when READSEQ [19] lacks the corresponding conversion capability for rich-sequence features such as complex location features. Specific programs allow to rapidly extract all reading frames from a given neighbouring genome (routine "Proteome extractor") or to translate a given EST or sequence read in all six reading frames (using the routine "6 frame translator"). The "Proteome extractor" extracts and assembles protein reading frames from GenBank and other primary databank records of a complete genome sequence. The routine uses the tag of "translation" and extracts directly all the protein sequences tagged like this from the genome data file (the complete predicted proteome). Moreover, we provide a solution to extract the coding proteins from unpublished sequence files; "6 frame translator" enables to select the sequence regions and translates these in all six reading frames.

To check in sequences with a function still unclear for encoded proteins the user can directly search with the nucleotide sequence in the COG database (program "COG master") as well as for different motifs (program "Pattern searcher"). "Pattern searcher" is complementary for sequence alignment and specialized for rapid pattern matching on protein and nucleotide sequences, in order to reveal functions by protein motifs and nucleotide elements. Prosite [20] motif-syntax and general regular-expressions are both supported. Bioperl modules [21] and translation by the "virtual ribosome" program [22] are used in parallel to predict and obtain proteins from EST cDNA data or variable sequence reads.

## Results and Discussion

For rapid EST or variable sequence read mapping, users upload a related genome template and the sequence reads/ESTs to be mapped. The rapid heuristic alignment algorithm in JANE compares sequences including interactive visualization in a genome browser. With the incorporated toolkit annotation information is readily obtained and the sequence can be eventually exported as part of the predicted new genome for further analysis (e.g. metabolism and other functions).

### Use case (i), mapping of ESTs

**Figure 2:**
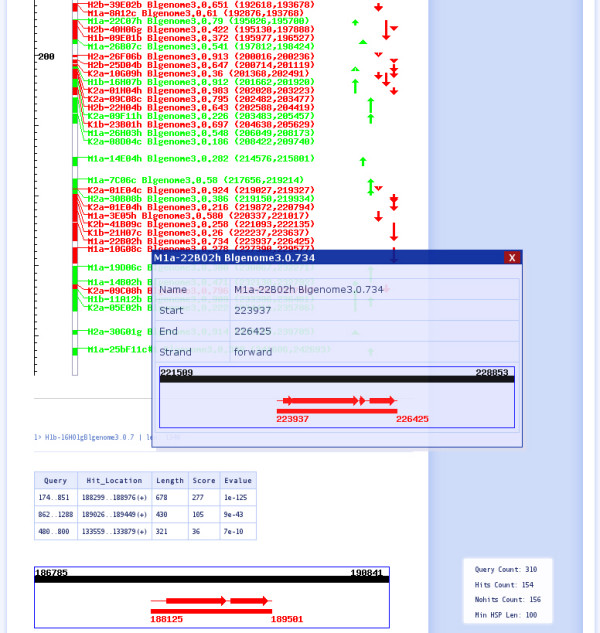
**Mapping of *Blattabacteria *short ESTs onto a moderate related genome template**. In the example, JANE maps a high fraction of ESTs to moderately related genomes (at least 80% rRNA identity/60% household enzyme similarity) with about 80% accuracy. The number in the left scale indicates the location in the genome template (in kilobases). All mapped ESTs are listed. Arrows in different colours (right) mark the consensus regions of mapping, i.e., red indicates the forward strand and green the reverse strand. A statistical report analyzes the mapping result (right corner). The inserts at the bottom show that by clicking on an individual EST detailed analysis is possible, the different HSPs used and their position appear. This includes also a list of all HSPs available as well as the chosen best EST match generated by JANE. Further analysis regarding all ESTs of that region, contig prediction, coding sequence and function prediction is also provided (see text). More details on the program options and an actual screen shot are given in Fig. S1.

Figure 2 illustrates how ESTs are mapped to common genome regions using a phylogenetically related genome template. In the example, ESTs from *Blattabacterium spp*. (symbiont from the cockroach *Blattella germanica*) are mapped by JANE using the related template genome of *Candidatus Sulcia muelleri *GWSS (previously sequenced, [23]). Evidently, sparse EST information is efficiently mapped and allows already with minimal information a first look at the arrangement of mapped ESTs/predicted transcription units. JANE starts with the template genome, and maps then ESTs from a new organism. Counters indicate which short reads or ESTs are not yet assigned, how many ESTs are currently assigned ("Hits") and how many short reads have too low score to be assigned ("Not assigned").

The template genome is step by step replaced by aligned short reads. Genome structure and genome features can be assessed already long before the sequencing effort of the genome is completed (predicted "virtual *Blattabacteria *genome"). In this EST mapping task using an only moderate (see below) related genome template (Fig. 2), the algorithm nevertheless allows to assign about 50% of all ESTs when thresholds of 10^-3 ^(E-value) and 100 bp (minimum length of HSP) are applied. Thus in the example given, an assignment applying BLAST with optimal settings (see above) for this task detects only 44 significant hits. Instead, applying JANE's heuristic algorithm, 154 ESTs from a total of 310 ESTs are successfully located.

We examined the mapping accuracy using a "fingerprint" test of COG hits. For each EST, a COG number match between the EST and the correct COG using the correct genome region is counted 1 point, each mismatch (wrong COG or KOG assigned) gets zero points. In the example, this returns for the *Blattabacterium *EST mapping using *Candidatus Sulcia muelleri *GWSS as template 80.0% accuracy of the mapped hits. Additional file 1 with its Fig. S2 illustrates EST mapping of genes for purine metabolism, applying a more distant genome template (*Gramella forsetii*). The annotation was verified as correct by subsequent sequencing.

We furthermore tested mapping of ESTs from different sources on various template genomes such as *Candidatus sulcia muelleri *[GenBank: CP000770], *Gramella forsetii *[GenBank: CU207366], *Staphylococcus aureus *N315, COL [GenBank: BA000018, CP000046] and *Staphylococcus epidermidis *ATCC 12228 [GenBank: AE015929]. For best results, the closest related genome available should in general be used as template. For detailed comparisons of potential template genomes we established a dedicated tool (InGeno [24]). However, a distance as far as *Gramella forsetii *or *Candidatus Sulcia *to *Blattabacteria *is sufficient as a solid basis for genome reconstruction with JANE. This corresponds to just more than 80% identity in the 16sRNA or more than 60% sequence identity in household enzyme protein sequences. Certainly this does not remove reconstruction problems which may arise from complex genome rearrangements or extended and short repetitive sequences in the new genome. However, fast mapping of a high fraction of the variable reads outside of such regions is nevertheless easily possible with JANE.

### Similar use case (i): rapid mapping of multiple sequencing reads

**Figure 3:**
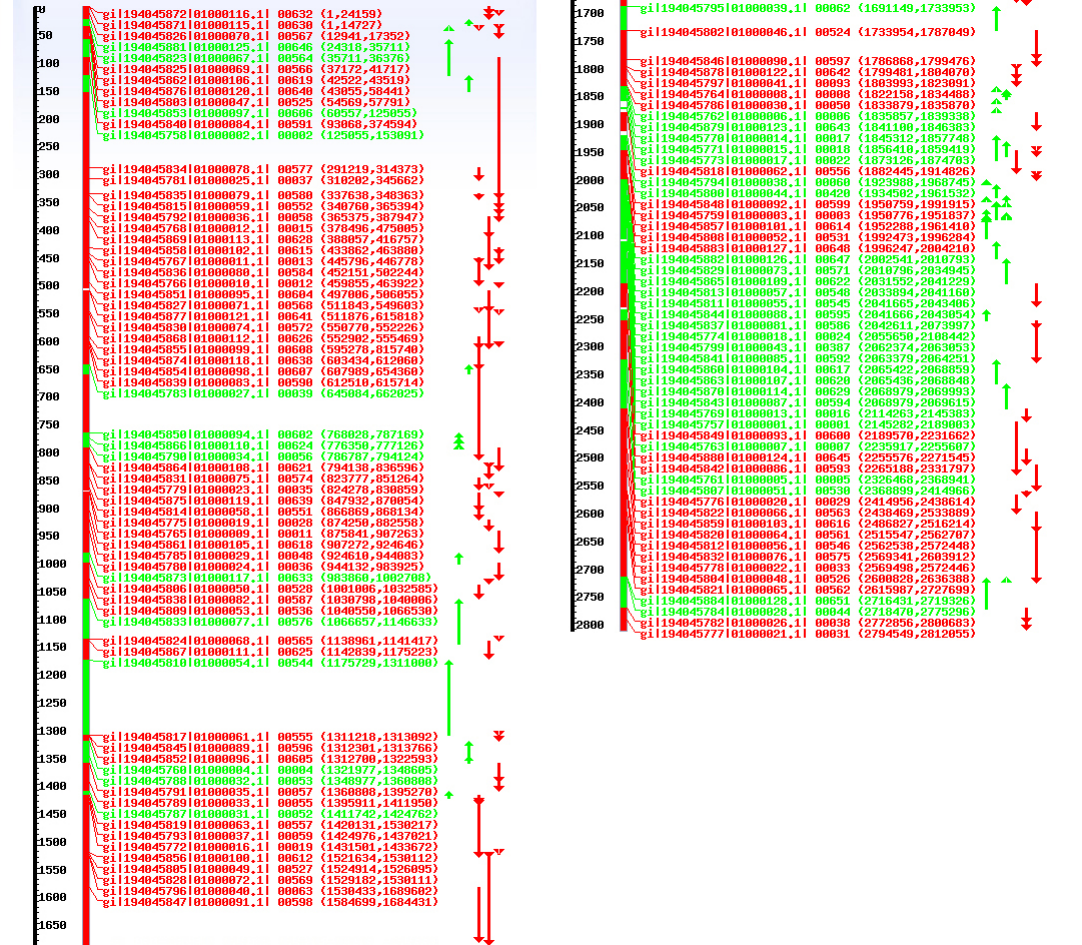
**Mapping of *S. aureus *JKD6008 contigs on *S. aureus *N315 genome**. Using a related template genome, rapid mapping of sequence reads, or of pre-assembled contigs is smoothly achieved. As in Fig. 2, the number in the left scale indicates the location in the genome template (in kilobases), arrows in different colours mark the consensus regions of mapping, i.e., red indicates the forward strand and green reverse. All mapped sequences are listed. Together, after mapping, they represent the major part of the JDK6008 genome.

Moreover, numerous sequence reads from pyrosequencing or other methods are rapidly mapped by JANE. An illustration example concerns *Staphylococcus aureus *JKD6008 (unfinished whole shotgun sequences) [GenBank: ABRZ01000001-ABRZ01000128]. This is a low-level vancomycin-resistant and persistent methicilin-resistent *S. aureus *isolate [25]. 128 JKD6008 contigs from GenBank were acquired and mapped on the nucleotide sequence of *Staphylococcus aureus *N315 genome (Fig. 3). Mapping quality according to COG fingerprinting (see above) is 92.10% and 125 contigs from the total of 128 can be mapped. We illustrate [see Additional file 1: Fig. S3] that using *S. aureus *N315 strain as template works equally well (correlation of correctly assigned COGs). JANE is able to deal with and assemble fairly short sequencing reads (20-40 nucleotides, e.g. SOLID, short ILLUMINA reads [26]) but shows for longer sequences (paired end ILLUMINA reads, 454 sequencing) best performance also compared to alternatives (see below).

### Comparison to Blast

Results for comparison of our EST mapping with a golden standard heuristic for sequence alignment, BLAST [14], are good. In particular, sensitivity of EST matches is improved significantly, allowing mapping even to distant template genomes. Nevertheless, specificity remains high in JANE, i.e. correct matching of ESTs to the template genome as examined for different template genomes. These results rely on the specific algorithm incorporated in JANE to optimally select and harvest HSPs, and are independent from an optimal parameter choice of the BLAST algorithm (which in our comparisons is of course already set with optimal parameters for generating potential HSPs in the EST mapping task).

### Comparison to EST alignment programs

We can furthermore compare the alignment results achieved to popular EST alignment programs. The software EST2Genome applies a Smith-Waterman algorithm in the first local-alignment pass to improve the sensitivity [27,28]. The quality of its alignment is similar to JANE's prediction if the template genome is the same genome from which the ESTs were derived. For more distant genome templates, ESTs are lost from mapping EST2Genome in the same way as in the above comparison to BLAST. Moreover, our program has considerable higher speed (in the example 8.7 seconds versus 1352 minutes). Exonerate [29], a successor to Genewise [30] has a similar speed as the JANE software when performing an ungapped alignment. Its est2genome alignment-model which considers gaps is relatively time-consuming and has advantages of accuracy for eukaryotic EST alignment. However, in mapping of distantly-related prokaryotic sequences, JANE prevails on both the number of located ESTs (see above) and the computation speed. EST2Genome and Exonerate are outstanding programs optimized for eukaryotic EST mapping, whereas JANE plays a critical role for rapid obtaining a mapping in particular for moderate-related prokaryotic sequences.

### Comparison to short sequence read assembly programs for ultrafast DNA sequencing

A recent focus of interest is ultrafast sequencing. Algorithms developed for this have the typical use case to align short reads. In the following we compare JANE to these as well as ultrafast sequencing algorithms for long reads as here much development is going on. Detailed results show reasonable performance for JANE but stress that this is not the optimal use case for JANE as it excels if reads have to be mapped to a non cognate genome. Thus two new developments, SOAP [7] and RMAP [10] appeared in 2008 as command-line tools just after the algorithm for JANE was finished. Both of them are designed to efficiently handle genome mapping of high-throughput short reads. The SeqMap tool arose recently, the command-line driven program offers various command-line options to give the highest number of mapped reads when dealing with short reads [8]. However, none of this alternative software has an interactive graphical interface allowing close up and distant views or separate views on start alignments and full alignments as JANE does. Table 1 compares the performance on contig mapping of Staphylococci (top), EST mapping of *Blattabacteria *(middle) as well as mapping very short reads to the cognate template genome (bottom). The short reads (bottom) are chopped ESTs, as these are not so often used, Table 2 and Table 3 deal with more typical data (Solexa reads, 454 reads) to test the ultrafast-sequencing software. The experiments were conducted in a computer with 4 GB RAM and an intel E6300 CPU running an Ubuntu 8.10 linux system. Local sequence files previously located in the JANE server were applied to avoid biased comparison by the effect of file-uploading time. RMAP is fastest among these programs, whereas SeqMap appears comparatively slow. The running time of JANE is relatively longer in comparison with other applications, however, this includes additional time for JANE's figure rendering procedure. Regarding longer sequences (Table 1 top), JANE is able to locate the highest number of reads (125 hits from a total of 128, i.e., 97.7%). SOAP is specialized for short sequence reads (20-40 nucleotides with read-length limits) and has large memory requirements but is very efficient (100%) and fast for short sequence mapping (Table 1 bottom). Another ultra-fast sequencing software is Maq [6], developed in 2007, which is particularly designed for the Illumina-Solexa genetic analyzer to efficiently align and assembly of high-throughput short reads (no longer than 63 bp) using a reference sequence (see example and manual extract in Additional file 1). It actually is the fastest program in the mapping task for short sequences to their cognate template (Table 1, bottom). However, its short read requirements make it not suitable to map longer reads or reads with variable lengths. For comparison, JANE prevails in dealing with complex situations or longer reads (Table 1 top, second rows) as it has a specific HSP harvesting and assembly algorithm. It is able to tolerate more substitutions, inversions, limited insertions and deletions in comparison to the compared programs. This grants JANE the capability to generate a virtual genome also from large-scale EST analyses and study these with the included function-prediction toolkit. We carried out a benchmark test on variable ESTs as well, 310 *Blattabacteria *ESTs were aligned to the *Candidatus Sulcia muelleri *GWSS genome (Table 1, second row). The results are summarized in Table 1. For a fair comparison, we tuned the parameter of maximum allowed mismatches for RMAP to 10 to increase its sensitivity. As a result it correctly maps 41.3% of the 310 ESTs. We enabled the maximum allowed mismatch parameter (= 5 max) of SeqMap, however, in spite of this and its strong performance in the direct mapping task (Table 1, bottom) it still can not locate any ESTs in this more complex situation. In the complex examples given, JANE is advantageously used to obtain best mapping results.

**Table 1:** Benchmark tests of different alignment software.

Application		RMAP	**SeqMap**^**8**^	**Maq**^**5**^	**SOAP**^**6**^	JANE	**Bowtie**^**7**^
Long reads (contigs)^1^	*Running time (s)*	*2.2*	*18.2*	*3.2*	*n.a*.	*15.7*	*9.6*
	
	*No of mapped reads*	*107* *(83.6%)*	*0*	*0*	*0*	*125* *(97.7%)*	*0*

**Variable ESTs**^**2**^	**Running time (s)**	**0.8**	**13.1**	**1.0**	***n.a*.**	**8.7**	**3.1**
	
	**No of mapped reads**	**128** **(41.3%)**	**0**	**0**	**0**	**154** **(49.7%)**	**0**

**EST fragments (40 bp)**^**3**^	**Running time (s)**	**0.4**	**7.8**	**1.7**	**2.4**	**8.1**	**3.3**
	
	**No of mapped reads**	**28** **(9.0%)**	**186** **(60.0%)**	**172** **(55.5%)**	**172** **(55.5%)**	**239** **(77.1%)**	**170** **(54.8%)**

Shortest reads (40 bp)^4^	Running time (s)	0.4	7.4	1.6	2.4	7.9	3.7
	
	No of mapped reads	310 (100%)	310 (100%)	310 (100%)	310 (100%)	310 (100%)	310 (100%)

We indicate the challenge in mapping and the distance of the template genome used for mapping. Bold: Moderate similar template genome, italics: closely-related genome template. Normal: cognate genome template.

^1^Long reads: 128 from a library of *Staphylococcus aureus *contigues (minimum lengths 627 bp or longer). Times are given in seconds. Accuracy of mapping was determined as given in materials and methods.

^2^Variable ESTs: 310 from a library of *Blattabacteria *reads with a minimum length of 19 bp.

^3^EST fragments trimmed to a fixed length of 40 bp.

^4^Short reads: 310 artificial fragments of 40 nucleotide length generated from the *Blattabacteria *genome sequence.

^5^Maq is not suitable for long reads (see Additional File 1), e.g., ESTs of variable lengths, it is customized for the Illumina-Solexa genome analyzer with a sequence limit of 63 bp.

^6 ^n.a. not applicable for mapping long reads due to a length limit of SOAP (specialized on very short reads of 20-40 bp).

^7^The alignment procedure of Bowtie is remarkable faster than other software, however for a fair comparison, the time for index building has still to be included.

^8^SeqMap did not find one entry when aligning short reads, but this is an untypical case with long repeats, we repeated the test replacing this read, the number of mapped reads is then 100%.

**Table 2:** Benchmark test on mapping Solexa reads

Application	Mapping reads* to chromosome 12 contig	Mapping reads* to chromosome 21 contig
RMAP	27 (2.7%)	10 (1.0%)
JANE	37 (3.7%)	18 (1.8%)
SeqMap	26 (2.6%)	7 (0.7%)
SOAP	37 (3.7%)	11 (2.0%)
Bowtie	37 (3.7%)	11 (2.0%)

* The total number of reads is 1000, we eliminated the incomplete or ambiguous reads in order to ensure all the programs run smoothly across the benchmark test. Both templates are *Homo sapiens *chromosome fragments within an acceptable length range for all the above applications (Solexa reads of 36 bp).

**Table 3:** Benchmark test on single-cell genome mapping^1^.

Application	Running time (s)	**No of mapped reads**^**2**^
**RMAP**	0.7	3 (0.65%)
**Maq**	3.3	0
**JANE**	7.1	103 (22.3%)
**Exonerate**	3980	n.a. ^3^

^1^Example: pori bacteria sequence reads are mapped to the template genome *Rhodopirellula Baltica *SH1. These are typical long reads (300 bp and more). ^2^Accuracy of mapping (fingerprint test) was determined as given in materials and methods. ^3^Exonerate assumes a eukaryotic genome with splicing. Splicing events are introduced by Exonerate if the sequence stretch is short and then an intron (several thousand base pairs) is assumed until the next local match. Thus, in the tough case above, all ESTs mapped (462 from 462) were sliced by Exonerate in several pieces and only short regions were aligned. If there were longer stretches they were mapped to the same region as JANE, but did have shorter length in the alignment.

#### Further detailed comparisons

In modern ultrafast sequencing use cases, the generated and often short (20-40 bp, typical 36 bp in several ultrafast sequencing methods) or longer fragments (e.g. 40-500 bp or even longer fragments in improved Solexa/SOLID or current 454 sequencing efforts) are mapped to the cognate template. A typical use case of mapping 36-400 bp long sequence reads to the correct DNA template (use case iii, ultrafast sequencing use case) is of course well handled by Solexa-specific software for ultrafast sequencing (Table 2). JANE's alignment is not originally designed for this purpose. It is relatively greedy and consumes longer time when aligning high-throughput reads. We applied sample files mapping Solexa reads on human genome contigs as given in additional files 2, including the sample files used for RMAP testing, which is to map Solexa reads to a contig of *homo sapien *chromosome 12. The first 1000 Solexa reads were uploaded to the server to align them to the genome template. Interestingly, JANE is able to locate more reads than RMAP or SeqMap does, however, RMAP runs much faster than JANE for mapping these Solexa reads. Both Bowtie and SOAP revealed 37 Solexa reads which were the same as found by JANE. In addition, we aligned these reads to another contig of chromosome 21, JANE was able to locate more reads in comparison to all the other software in Table 2. All mapped reads were re-examined to be highly significant matches (e < 10^-6^).

A key advantage of JANE compared to ultrafast sequencing mapping algorithms is its capability to deal with complex use cases where fragments of variable length are mapped to a non-identical reference either in EST mapping or in single cell sequencing as the genome sequence is either not yet or never fully known. In order to acquire a comprehensive benchmark, we used for one set of comparisons a set of 310 short sequences, all of them generated from the *Blattabacteria *genome with a fixed length (40 bp; the size all programs can accept; some programs require only short reads). These are clearly short sequence reads and all the software returns an outcome of almost 100% hits during the alignment. The sequences are first mapped to their own genome template (bottom row in Table 1). In this task, RMAP [10] is the fastest, Maq [6], SOAP [7] and Bowtie [9] are second, however, all the programs are able to complete the jobs within an acceptable time. In addition, focusing only on the alignment procedure, bowtie runs most rapid among all applications we compared. However it requires a particular index-building procedure, this additional time requirement has to be considered in addition (around 3-5 seconds depending on the complexity of the data to be indexed).

Next we did a similar but slightly more complex mapping test using now short EST sequences (trimmed to 40 base pairs). We mapped these now onto a related genome, *Candidatus Sulcia muelleri *GWSS (top row in table 1). Here RMAP is the champion only regarding execution time but SeqMap, Maq and SOAP mapped more ESTs in comparison to RMAP. JANE performed best on locating them during the test. Finally, mapping typical ESTs of variable lengths to a related genome is not suitable for all algorithms. In this more challenging test (bold, middle row in table 1), only RMAP and JANE are able to locate them and return the mapping results. Similar to this we give data on an experiment of mapping long contigs (italics, top row in table 1). Here JANE successfully locates the highest number of reads, followed by RMAP.

### Use case (ii), single cell sequencing tasks

JANE is particularly advantageous if variable sequence reads between few base pairs (>20) up to thousand base pairs have to be mapped to a non-identical genome template. This occurs typically in single cell sequencing [5], an upcoming method to obtain genome sequences from individual, non cultivable bacteria [4]. An example from our own work (Table 3) involves mapping of pori-bacteria sequence reads, a new bacterial phylum resident in porifera. The closest available relative with a complete genome sequence is *Rhodopirellula Baltica *SH1, already a phylum away (so as demanding as e.g. mapping *E. coli *sequence reads onto *B. subtilis *genome sequence). Table 3 summarizes mapping results of 462 pori-bacterium reads from a single cell sequencing experiment mapped onto this template. The sequences are typical long reads (454 reads, Sanger reads). We apply 100 bp as the minimum alignment width during the benchmark test to minimize false hits and allow comparison across different programs. RMAP and Maq execute relatively faster with fewer hits located, however JANE reveals more successfully mapped regions beyond the cutoff minimal alignment length. This is understandable, since RMAP, Maq, SeqMap and SOAP are designed for rapid alignment of short reads such as Solexa reads, whereas the algorithm of JANE allows to map a wide range of sequence read lengths (few base pairs to kilo bases). Here Exonerate is not really comparable as it is trained and used for eukaryotic genomes. Thus it takes 66 minutes to exhaustively search the genome and mapped regions are always disturbed by spurious predicted intron regions. For this prokaryotic single cell sequencing use case, JANE performs best in the task and maps 103 (22.3% out of all the fragments, evaluation by fingerprint test as described above). This 22.3% apply for the first mapping iteration. If the next single cell of a pori bacterium is sequenced, the JANE-optimized template genome (where then already parts of *Rhodopirellula Baltica *have been replaced by the pori bacterium) may be used and thus more and more sequence reads are successfully mapped in later iterations, provided the poribacteria are picked from the same strain.

#### Further comparisons

High throughput pyrosequencing is now able to provide sequence reads of over 400 bp, further tests on JANE were carried out on these long *de novo *sequences (454; Sanger reads). For this case, Roche's Newbler assembly software is in principle able to tackle the aligning and assembly problem since it is particularly designed for the 454 system, however, it requires a commercial license so it could not be used for our benchmark test. Another popular application is the phred and phrap package [31], which performs efficiently when assembling shotgun reads and incorporates quality data. However it lacks the feature to map the reads from 454 or Solexa techniques to a reference template such as RMAP, Maq and SeqMap do. Of course, if quite compact (viral genome), you could treat the genome as another very long read but this requires excessive memory studying bacterial genomes and is not practical (data not shown).

Phrap is designed as a *de novo *assembler which is not suitable for the orthology mapping or assembly with a backbone genome, thus we did not include this software in our comparisons. However, there is a consecutive application "consed", which allows aligning reads to a reference sequence, but here again only the identical template is used. The comparisons show short sequence alignment software prevails in efficiency in particular when aligning high throughput reads, e.g., more than 10,000 entries which online software can not afford. Ultrafast sequencing and mapping to the cognate template is thus not the optimal use case for JANE, however JANE's algorithm provides relatively higher sensitivity and locates the same number of or more reads with an acceptable speed. In particular, JANE offers here a very simple standalone approach for single cell sequencing including mapping to more or less related genomes. Results can then later be complemented by more sophisticated and demanding approaches.

### Comparison to related programs offering genome viewers and function assignment

Concerning function assignments, CAMERA [32] offers a comprehensive platform for ecology research and analysis, its fragment recruitment viewer illustrates the spread of metagenomic sequence reads across species as an encyclopedia. It provides interesting views for comparative metagenomic studies and related-functions are revealed. Similarly, the MG-RAST server [33] offers a pipeline to assist researchers to acquire a rapid functional assignment for sequences of the metagenome by comparing both translated protein and nucleotide databases. Similar and related programs are the RAST server [34] and IMG/M server [35]. However, in addition to this software, JANE is able to deal with fragment assembly, both for short oligonucleotide sequences and longer reads and rapidly predicts the putative genome regions which guide the next iteration of sequencing.

JANE provides a platform for achieving a rapid impression of genome structure and gene functions. Cap3 provides an extensive precise sequence assembly and correction procedure [36], but has no mapping option for ESTs. Combining JANE and Cap3 is possible and should boost sequencing progress. Finally, there is the recent development "Circos", a powerful graphical figure generator for genome comparisons and to render results as various types of circles using different Perl scripts [37]. There is no graphical user interface and the output is specifically intended to be non-linear. Also this tool can be well combined with JANE in prokaryotic transcriptome projects.

## Conclusion

JANE allows rapid mapping and assembly of ESTs and variable length sequence reads also on non-identical, closer or more distantly related genome templates. It outperforms in this task alternative algorithms. Mapping is thus independent from whether the genome sequence of the prokaryote in question becomes available. This is important in single cell/*de novo *sequencing (complete genome is not available including even non-cultivatable bacteria) and RNA-based transcriptome studies (either before the full genome sequence is available or without a genome sequence, e.g. in clinical isolates). In addition, JANE includes function prediction and quality control of mapped ESTs/reads, is standalone, easy to setup and open source including the source code (publicly available at the website).

## Availability and requirements

• Project name: **JANE**

• Project homepage: http://jane.bioapps.biozentrum.uni-wuerzburg.de

(Example sequences and program download are available in the querying page.)

• Programming language: Perl

• Operating systems: Web-application available platforms, Windows, Linux, General Unix, Macintosh.

• License: free for non-commercial use; use for non-academics: contact corresponding author.

## Authors' contributions

CL: programming and testing of JANE, writing of the ms; AS: testing of JANE, mapping of sequence reads, EST function analysis; MLS: genome sequencing, EST sequencing, analysis of ESTs and genome; AM: expert advice on genome projects, supervision of MLS; RG: expert advice on microbiological EST mapping in *Blattabacteria *and single cell sequencing; JB: expert advice on software development; TD: advice, organisation and guidance of the study, testing of JANE, writing of the ms; All authors read and approved the final manuscript.

## Supplementary Material

Additional file 1 **Three Figures, their legends and a text**. Further results, supporting material Click here for file

Additional file 2 **solexa reads of human genome, homo sapien chromosome 12 contig, chromosome 21 contig**. sample files for testing the mapping of Solexa reads tests; mismatches allowed are 3 for all software and the e-value threshold is 0.00001. Click here for file
