# Supplementary material for: JANE: efficient mapping of prokaryotic ESTs and variable length sequence reads on related template genomes
Source: BMC Bioinformatics. 2009 Nov 29;10:391. doi: 10.1186/1471-2105-10-391 (PMC2789075; doi:10.1186/1471-2105-10-391)
Supplement: Additional file 1 — Three Figures, their legends and a text. Further results, supporting material [file 1471-2105-10-391-S1.doc]

**Supplementary material**

**Fig.S1 JANE functionalities (example: mapping of *Blattabacteria*** **short ESTs onto a moderate related genome template).** This is a screenshot from the JANE program. On top options for the users include (left to right) input file choice, documentation of help and tutorials. Next follow tools for function annotation (patterns and COGs), format conversion, proteome extraction and codon translation (screen will change always accordingly). Staff denotes people involved in the project, however, as the “Login” and “Register” button this can be adapted for any ongoing sequencing or annotation project. As in Fig. S1, JANE maps a high fraction of ESTs to moderately related genomes (at least 80% rRNA identity / 60% household enzyme similarity) with about 80% accuracy. The number in the left scale indicates the location in the genome template (in kilobases). All mapped ESTs are listed. Arrows in different colours (right) mark the consensus regions of mapping, Red indicates the forward strand and green the reverse strand. Links to other databases or resources are listed in the right panel. The insert at the bottom shows that by clicking on an individual EST detailed analysis is possible, the different HSPs used and their position appear. Further analysis regarding all ESTs of that region, contig prediction, coding sequence and function prediction is also provided (not shown, see text).


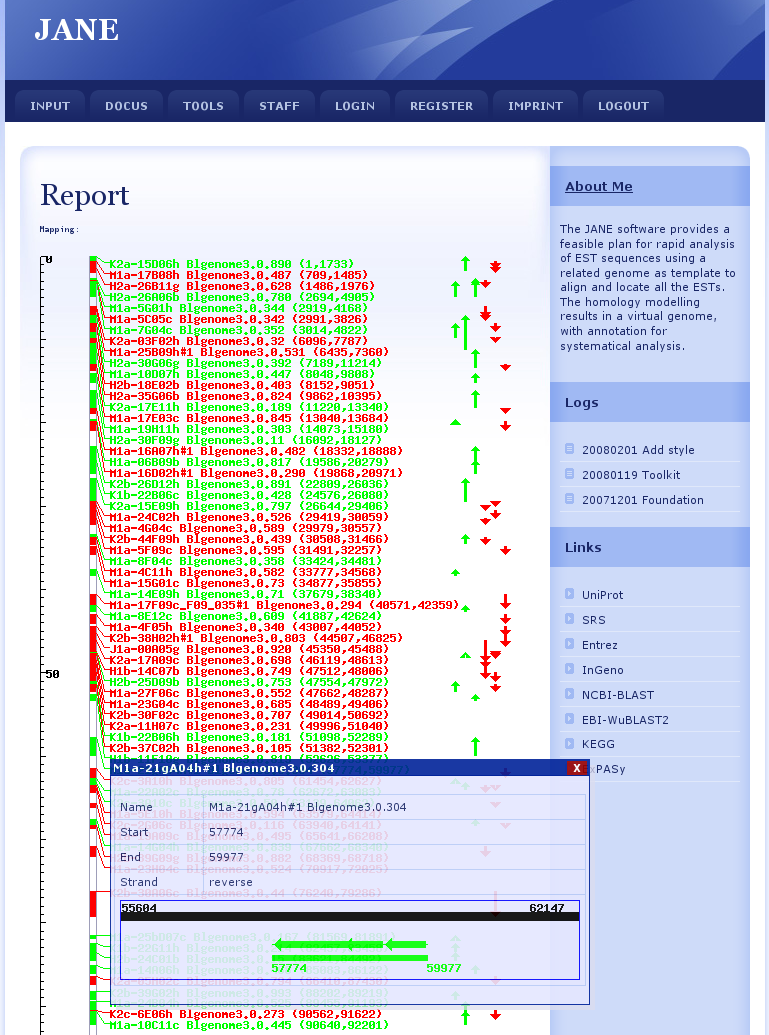


**Fig.S2 EST mapping.** Even if only very few mRNAs or ESTs are known from a new genome, the template genome approach applied by JANE allows their rapid mapping and predicts their neighbourhood, the example shows two RNAs encoding purine metabolizing enzymes. The insert shows accurate mapping and coverage, the bar on the left shows the distances estimated according to the template genome (example: *Blattabacteria* ESTs on the template genome of *Gramella forsetti*).

**
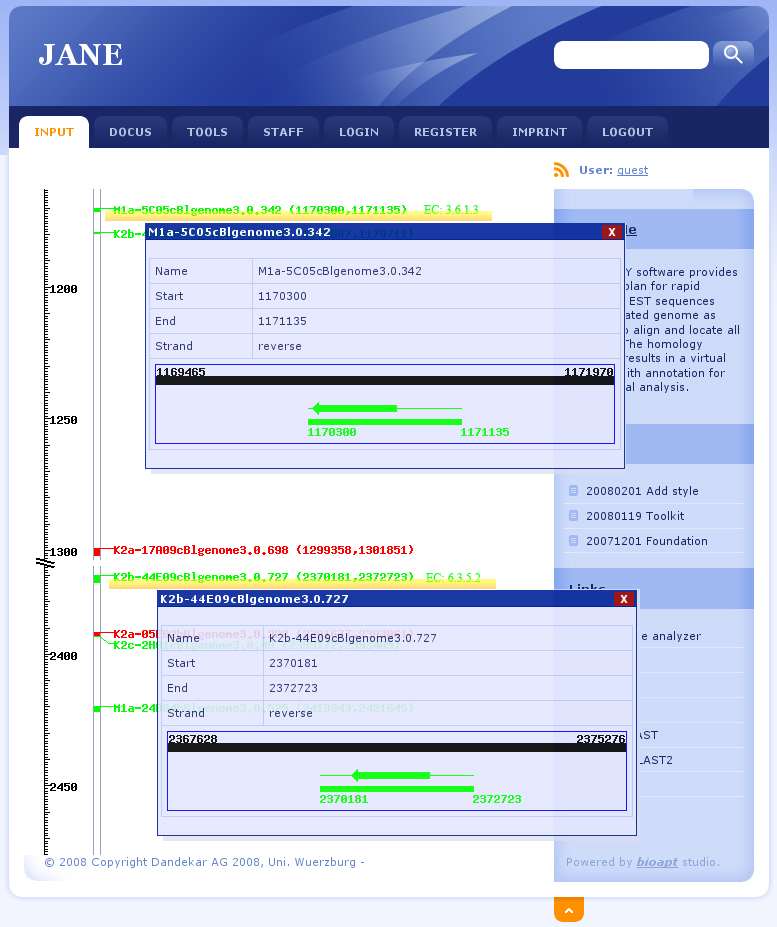
**

**Fig.S3 Pairwise comparison of mapping results between two close-related genome template using R.** The x-axis indicates the mapping positions of fragments in the *Staphylococcus aureus* COL genome, the y-axis the locations in the N315 genome. The dot located near the putative regression line, the diagonal in the figure, suggests the contig region is highly-conserved and the algorithm gives a definite position. The method is used in addition to the “COG fingerprinting” method for verifying the accuracy of the alignment.

**
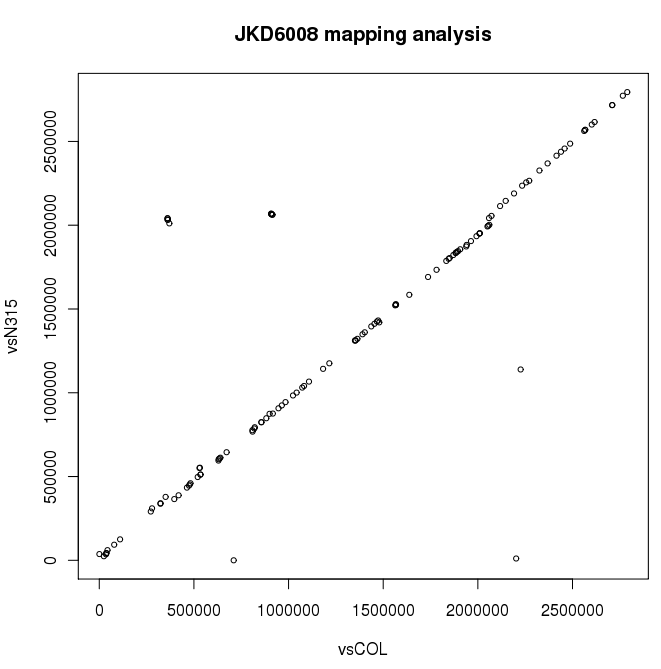
**

**Maq description from online manual (http://maq.sourceforge.net/maq-manpage.shtml#2).**

Maq is a software that builds mapping assemblies from short reads generated by the next-generation sequencing machines. It is particularly designed for Illumina-Solexa 1G Genetic Analyzer, and has a preliminary functionality to handle AB SOLiD data.

With Maq you can:

o Fast align Illumina/SOLiD reads to the reference genome. With the default options, one million pairs of reads can be mapped to the human genome in about 10 CPU hours with less than 1G memory.

o Accurately measure the error probability of the alignment of each individual read.

o Call the consensus genotypes, including homozygous and heterozygous polymorphisms, with a Phred probabilistic quality assigned to each base.

o Find short indels with paired end reads.

o Accurately find large scale genomic deletions and translocations with paired end reads.

o Discover potential CNVs by checking read depth.

o Evaluate the accuracy of raw base qualities from sequencers and help to check the systematic errors.

However, Maq can NOT:

o Do de novo assembly. (Maq can only call the consensus by mapping reads to a known reference.)

o Map shorts reads against themselves. (Maq can only find complete overlap between reads.)

o Align capillary reads or 454 reads to the reference. (Maq cannot alig
